# Revisiting the co-crystal structure of a DNA glycosylase with photocaged substrate: a suitable time-resolved crystallography target?

**DOI:** 10.1107/S2052252525006062

**Published:** 2025-07-14

**Authors:** Tomoki Imura, Yuhei Hosokawa, Kai-Chun Yang, Yuki Ban, Hsuan-Yu Shih, Junpei Yamamoto, Manuel Maestre-Reyna

**Affiliations:** a Division of Chemistry, Graduate School of Engineering Science University of Osaka 1-3 Machikaneyama, Toyonaka Osaka560-8531 Japan; b Department of Chemistry National Taiwan University 1 Roosevelt Road, Sec. 4 Taipei106 Taiwan; c Institute of Biological Chemistry Academia Sinica 128 Academia Road, Sec. 2 Taipei115 Taiwan; University of Melbourne, Australia

**Keywords:** DNA repair, photocaged substrate analogs, hOgg1, intermediate trapping

## Abstract

Co-crystal structures of a base-excision DNA-repair enzyme (human 8-oxo­guanine DNA glycosylase; hOgg1) in complex with a photocaged 8-oxoguanine DNA lesion were determined before and after uncaging via illumination at 2.81 and 2.48 Å resolution, respectively. The structures were carefully reassessed to rapidly expand the target repertoire of light-triggered time-resolved macromolecular crystallography. Late-intermediate cryo-trapping after uncaging revealed the partial accommodation of 8-oxoguanine in the active site with 68% occupancy, which did not induce full active-site adaptation to the catalytic state. Crystal illumination led to a light-dependent loss of diffraction power, likely due to crystal-packing collapse during the very late reaction stages. This work therefore not only demonstrates that hOgg1 is well suited for time-resolved crystallography, but also that such analysis is necessary to determine further steps in its reaction.

## Abstract 2

Engineered *in crystallo* photosensitivity in a DNA-repair enzyme is carefully assessed for its suitability as a target for time-resolved crystallographic studies.

## 1. Introduction

Over the past decade, advances in X-ray generation (Fromme, 2015 ▸; Spence, 2017 ▸; Milne *et al.*, 2017 ▸; Yabashi *et al.*, 2015 ▸) and detection (Kameshima *et al.*, 2014 ▸; Mozzanica *et al.*, 2018 ▸) have ushered in an age of enzyme cinematography (Maestre-Reyna *et al.*, 2023 ▸; Sorigué *et al.*, 2021 ▸; Kupitz *et al.*, 2014 ▸). Short-lived reaction intermediates can now be structurally characterized at atomic and femtosecond resolutions via light-triggered time-resolved crystallography (LT-TRX; Schmidt, 2019 ▸; Grünbein *et al.*, 2020 ▸), provided that large quantities of crystals can be produced. Light represents the ideal trigger for TRX experiments, as high temporal and spatial resolution necessitates synchronous reaction evolution across the crystal (Poddar *et al.*, 2022 ▸). Photoenzymes, although rare in nature, have therefore been highly attractive targets for TRX analysis (Poddar *et al.*, 2022 ▸; Monteiro *et al.*, 2021 ▸). Conversely, studies on standard, light-independent enzymes have lagged because achieving their uniform *in crystallo* activation is very challenging (Monteiro *et al.*, 2021 ▸). For example, mix-and-inject TRX (MI-TRX) has mostly been confined to millisecond resolution, as diffusion of the substrate through the crystal often becomes the rate-limiting step of the reaction (Schmidt, 2013 ▸).

Photocaged substrate analogues (PSAs) may overcome the limitations imposed by *in crystallo* substrate diffusion (Monteiro *et al.*, 2021 ▸). PSAs contain a photolabile protecting group which prevents the formation of the substrate–enzyme complex, with deprotection occurring upon illumination (Wang, 2013 ▸; Li *et al.*, 2023 ▸). Enzyme crystals could then be soaked in a PSA solution in the dark, after which the reaction is monitored via LT-TRX. Since PSAs have already pervaded the crystal lattice upon triggering of the reaction, substrate diffusion becomes less of a factor and reaction intermediates become kinetically resolvable (Monteiro *et al.*, 2021 ▸; Mehrabi *et al.*, 2019 ▸).

**Figure 1:**
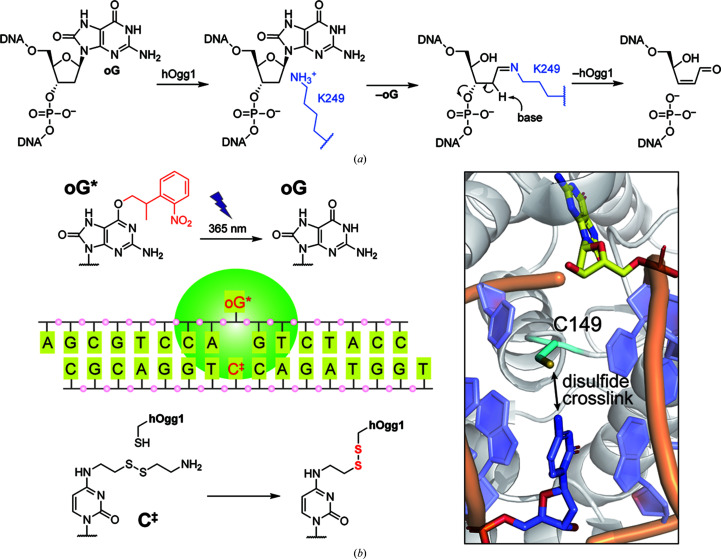
Reaction schemes of the oG/oG*–hOgg1 complex. (*a*) Base excision of oG by hOgg1 followed by DNA cleavage. (*b*) The reported oG*–hOgg1 system. The bulky *O*^6^-NPP group prevents the entry of oG* into the active site of hOgg1. Uncaging of oG by near-UV light triggers accommodation of oG in the active site, followed by a base-excision reaction. For crystallization, the oG*–hOgg1 complex was stabilized by a covalent bond. The cytosine base modified at the *N*^4^ position with cystamine (C^‡^) complementary to oG* is cross-linked with the Cys149 side chain of hOgg1-N149C via a thiol–disulfide exchange reaction.

Establishing a PSA for a new enzyme target is not trivial. The kinetics of deprotection need to be carefully considered, as slow deprotection may become the rate-limiting step (Wang, 2013 ▸; Monteiro *et al.*, 2021 ▸). Additionally, a low-quantum-yield PSA will result in low activation within the crystal, and thus no significant TRX data may be obtained. Conversely, adapting previously existing PSA–enzyme pairs for LT-TRX experimentation is a viable strategy for the rapid development of working light-independent enzymatic model systems. One such pair is photocaged *O*^6^-(*o*-nitrophenisopropyl) 8-oxoguanine (*O*^6^-NPP oG, abbreviated oG*) and the human oG glycosylase 1 (hOgg1) (Lee *et al.*, 2008 ▸). As a DNA glycosylase, hOgg1 is a key part of the human base-excision DNA-repair pathway, and its mechanisms have been extensively studied both functionally and structurally (Banerjee *et al.*, 2005 ▸; Bruner *et al.*, 2000 ▸; Qi *et al.*, 2009 ▸; Shigdel *et al.*, 2020 ▸). hOgg1 is capable of recognizing the representative DNA lesion oG (Fromme & Verdine, 2004 ▸) by first binding potentially damaged DNA and then extruding the corresponding base from the DNA double strand, which results in formation of the G-interrogation complex (GIC; Shigdel *et al.*, 2020 ▸; Banerjee *et al.*, 2005 ▸). GIC is not catalytically competent, as the potential base is hosted within an exosite (Lee *et al.*, 2008 ▸). Only upon recognition of damage at the GIC stage is the nucleotide loaded into the active site, where oG was first excised, followed by cleavage of the DNA backbone via β-elimination [Fig. 1 ▸(*a*)] (Fromme & Verdine, 2004 ▸; Bruner *et al.*, 2000 ▸). Thus, in the strategy designed by Lee and coworkers, which relies on the crystallization of fully active, cross-linked oG*–DNA–hOgg1 complexes (Lee *et al.*, 2008 ▸), steric hindrance due to the photolabile protecting group in oG* prevented active-site loading, arresting the reaction cycle at the GIC stage. Subsequent illumination at 365 nm followed by flash-freezing cryotrapped a late reaction intermediate [Fig. 1 ▸(*b*)] (Lee *et al.*, 2008 ▸). In this work, we have adapted the oG*–hOgg1 system for work under TRX conditions. Here, we have (i) successfully scaled up both substrate and protein production, (ii) carefully analysed in-solution and *in crystallo* light-dependent reactivity, (iii) established high-throughput hOgg1–DNA co-crystallization conditions necessary for TRX methodologies and (iv) produced dark-adapted and light-triggered structures, which we have made available for the first time in the Protein Data Bank. Overall, we showcase an enzymatic system ready for LT-TRX analysis, and precisely characterize a cryo-trapped intermediate in the hOgg1 reaction cycle.

## 2. Materials and methods

### 2.1. Preparation and crystallization of a DNA–protein complex with a disulfide linkage

For crystallization, oligonucleotides containing oG* and the *N*^4^-modified cytosine (C^‡^), in addition to wild-type and N149C His-tagged hOgg1 proteins, were prepared as described in the supporting information. The following procedures were performed under dim red light. The damaged strand containing oG* (40 nmol) was hybridized to the complementary strand containing C^‡^ (40 nmol) in 400 µl buffer (10 m*M* Tris–HCl, 50 m*M* NaCl, 1 m*M* EDTA pH 7.5) by heating at 80°C for 5 min and cooling overnight. The double-stranded DNA (12 nmol) was then incubated with the N149C mutant (24 nmol) in 1.2 ml reaction buffer (20 m*M* Tris–HCl, 100 m*M* NaCl, 1 m*M* EDTA pH 7.4) on ice overnight. The DNA–protein complex connected via a disulfide linkage was purified by anion-exchange chromatography using a HiTrap Capto Q column, followed by size-exclusion chromatography using a Superdex 200 Increase 10/300 GL column and elution buffer (20 m*M* Tris–HCl, 1 m*M* EDTA, 100 m*M* NaCl pH 7.4). The concentration of the protein was determined by the Bradford assay and the complex solution was prepared at 13 mg ml^−1^ for crystallization.

Crystallization was carried out via the sitting-drop vapour-diffusion method at 4°C. Reservoir solutions [400 µl; 100 m*M* sodium cacodylate, 8–20% either polyethylene glycol (PEG) 6000, 8000 or 10 000, 100–300 m*M* of either CaCl_2_ or MgCl_2_ pH 5.5–6.5] were placed in the wells and 1 µl of the DNA–protein complex solution was placed on the post and mixed with 1 µl of each reservoir solution. The plates were sealed and placed for 2–3 days to yield rod-like crystals. In our screening, crystal showers were preferentially obtained at concentrations of PEG and metal ions higher than 12% and 100 m*M*, respectively, and at a pH higher than 6, regardless of the type of PEG and metal ion.

## 3. Results and discussion

### 3.1. In-solution characterization of DNA and hOgg1

**Figure 2:**
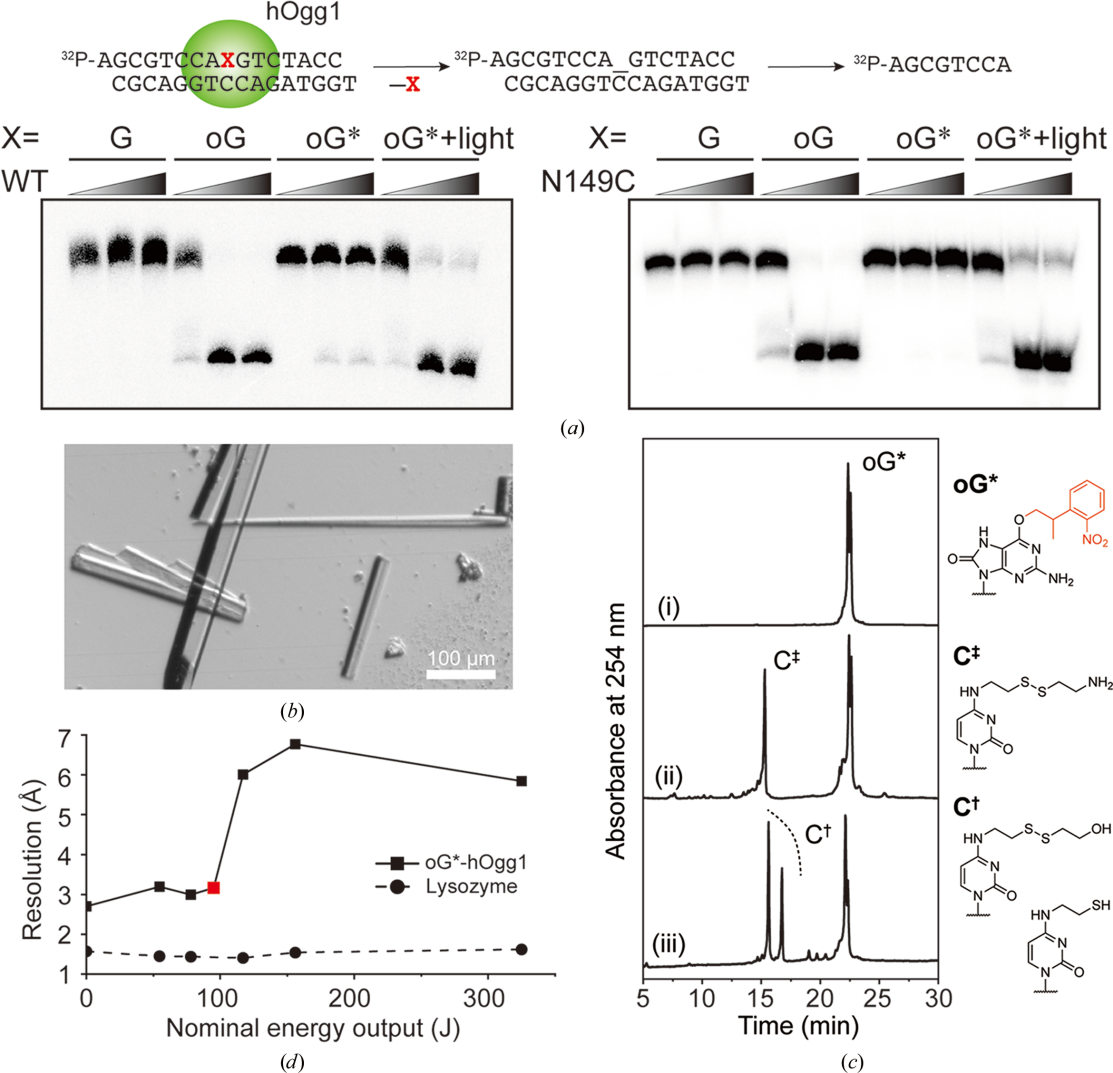
Verification of the oG*–hOgg1 system in solution and in crystals. (*a*) Enzymatic activity of WT and N149C hOgg1 with G-, oG- or oG*-containing oligonucleotides. The double-stranded DNA was incubated with 0, 1 or 2 molar equivalents of WT or N149C hOgg1. (*b*) A representative picture of G*O co-crystals. (*c*) HPLC chromatograms of (i) oG*-containing oligonucleotide, (ii) double-stranded DNA before complex formation and (iii) double-stranded DNA recovered from the crystals. (*d*) Diffraction power of G*O (square data points, solid line) and lysozyme (circular data points, dashed line) crystals versus 365 nm illumination dose. The red square represents the dose used to obtain the G*O_light_ data set.

In the reported oG*–hOgg1 system, the protein–DNA complex was stabilized via the introduction of a disulfide linkage between an engineered cysteine (hOgg1-N149C) and the *N*^4^-modified cytosine (C^‡^) complementary to oG* [Fig. 1 ▸(*b*)]. The complex was then crystallized in the dark. To adapt the system for use in LT-TRX, we prepared DNA substrates (Supplementary Table S1), as well as wild-type (WT) and N149C mutant hOgg1. Firstly, we verified in-solution photodeprotection of the caged oligonucleotide. Upon illumination with 365 nm LED light, the phenisopropyl group was removed and the oG-containing oligonucleotide was recovered as reported previously (Supplementary Fig. S1). The enzymatic activities of the WT and N149C mutant were also examined. In the dark, incubation of oG-containing DNA with the WT protein produced a shorter, oG-excised oligonucleotide. Meanwhile, base excision of oG* in the dark was prohibited by the presence of the photocaging group [Fig. 2 ▸(*a*)]. Upon illumination of the reaction mixture containing the photocaged substrate with 365 nm LED light the shortened product was observed, indicating that the activity of hOgg1 was restored by liberating the native oG from the bulky photocaging group. The same experiments were performed with the N149C mutant and the band patterns for the mutant were comparable to those for the WT, thus safely excluding the possibility that the mutation interfered with the catalytic activity of hOgg1. These experiments comprehensively support the capability for light-triggered hOgg1 reaction in the oG*–hOgg1 system.

### 3.2. *In crystallo* characterization

After hybridization of the oG*-containing oligonucleotide (Entry 3 in Supplementary Table S1) to the C^‡^-containing complementary strand (Entry 5 in Supplementary Table S1), the duplex was allowed to react with hOgg1-N149C. The covalent protein–DNA complex was successively purified via anion-exchange and size-exclusion chromatography, followed by crystallization under conditions optimized from a previous report (Lee *et al.*, 2008 ▸), reproducibly yielding oG*–hOgg1-N149C (G*O) co-crystals [Fig. 2 ▸(*b*)].

*In crystallo* oG* stability was tested with month-old co-crystals which had been kept in the dark. These were dissolved in water supplemented with 2-mercaptoethanol to cleave the disulfide bond between the DNA and protein. The recovered DNA was analysed by HPLC [Fig. 2 ▸(*c*)]. Clearly, the recovered DNA contained oG* in addition to the *N*^4^-modified cytosines (C^†^) without observing the uncaged oG oligo­nucleotide, showing the integrity of the DNA in the G*O co-crystals.

Crystal photosensitivity was tested by exposing fragmented crystals to 365 nm LED light for 10 s at varying energy doses, followed immediately by flash-cooling and data collection. Here, the exposure of G*O co-crystals to light clearly affected the crystal diffraction power [Fig. 2 ▸(*d*)], with dark crystals diffracting to ∼2.7 Å resolution. Crystals whose 365 nm light dose was below 100 J diffracted only slightly worse (∼3.16 Å). Crystals diffracted dramatically less on greater exposure to near-UV light, with no detectable diffraction at all beyond 300 J. As a control, lysozyme crystals of similar size were treated in the same way as the G*O co-crystals, but showed no photosensitivity at all [Fig. 2 ▸(*d*)], suggesting that light exposure caused the loss of diffraction power in G*O, rather than dehydration or other effects derived from crystal handling. The observed illumination-dependent loss of diffraction power in G*O is reminiscent of our previous study on light-driven DNA repair by a class II photolyase (Maestre-Reyna *et al.*, 2023 ▸), where light-induced, large-scale conformational changes in the protein–DNA complex disrupted crystal packing during the very late reaction stages. Indeed, the G*O crystal lattice also involves DNA-mediated crystal contacts (Supplementary Fig. S1). Considering the enzymatic activity of hOgg1, repair-induced *in crystallo* complex rearrangements may also underlie the observed loss of diffraction power.

### 3.3. Resting co-crystal structure of the oG*–hOgg1 complex

Prior to reaction initiation, the structure of the oG*–hOgg1-N149C complex (G*O_dark_) was solved at 2.7 Å resolution (Table 1 ▸, PDB entry 9kky). G*O_dark_ closely resembled previously published co-crystal structures of the hOgg1 GIC [Supplementary Fig. S3*a*)]. As a member of the oxidative DNA damage-repair enzyme superfamily (ODR, InterPro ID IPR052054), hOgg1 consists of an N-terminal OGG_N domain followed by a C-terminal helix–hairpin–helix-Gly/Pro-rich loop (HhH-G/P) domain, with only the latter making contacts with the double-stranded DNA (dsDNA) substrate [Supplementary Fig. S3(*a*)]. Interactions between the protein and the dsDNA are dominated by the Cys149 disulfide cross-link [Supplementary Fig. S3(*b*)]. Other interactions were limited in number, mostly occurring either at the lesion site [Supplementary Fig. S3(*b*)], where Tyr203 and Arg204 stabilized the unpaired bubble left due to partial oG* extrusion, or along the downstream branch [Supplementary Fig. S3*a*)] via the G/P-rich loop of hOgg1. Therefore, it is hardly surprising that the DNA remains largely dynamic in the complex, resulting in unusually high *B* factors around the refined DNA region of the G*O_dark_ structure (Table 1 ▸) and with no assignable electron density (ED) for the entirety of the upstream DNA arm or the oG* base moiety [Supplementary Figs. S3(*a*) and S4(*a*)]. The latter represents a significant difference when compared with the original GIC, where guanine was sandwiched between His270 and Asp268 at an exosite located near to the catalytic centre of the protein [Supplementary Fig. S4(*b*)]. Here, it is reasonable to assume that the bulky *O*^6^-NPP group which prevents the entry of oG into the active site also precludes binding to the exosite.

### 3.4. Structure of cryotrapped intermediate

**Figure 3:**
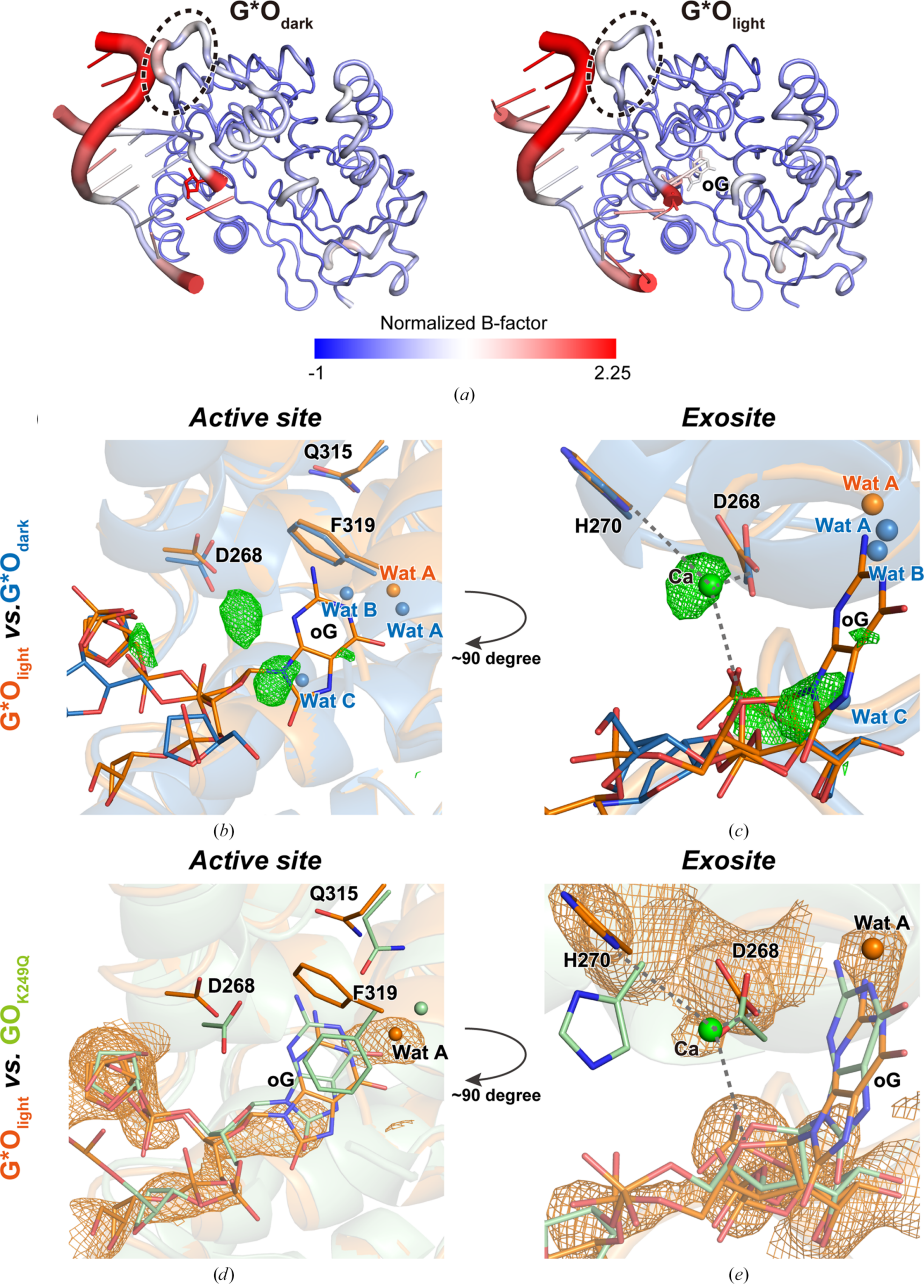
Structural features of G*O complexes. (*a*) Global side-by-side comparison of G*O_dark_ (left) versus G*O_light_ (right). DNA and protein are shown in a tube representation, with tube thickness proportional to normalized *B* factor. Structures are also coloured by normalized *B* factor, with the colour scale below the figure. *B* factors were normalized following the scheme described previously (Carugo, 2022 ▸). An oval discontinuous line marks the position of the G/P-rich loop. (*b*) Comparison of the G*O_dark_ (blue) versus the G*O_light_ (orange) active site. DNA/protein residues are shown as stick diagrams, while water molecules corresponding to G*O_dark_ are shown as blue spheres and those for G*O_light_ as orange spheres. A 3σ-contoured σ_A_-weighted *F*_o_(G*O_light_) − *F*_c_(G*O_dark_) DED map is superposed over the structural models. Positive DED peaks are shown in green; no negative peaks could be visualized. (*c*) Detail of the exosite of G*O_dark_ versus G*O_light_ shown as in (*b*). (*d*) Detail of the active site of G*O_light_ (orange) versus the previously published, catalytically inert, GO_K249Q_ complex (green, PDB entry 6w0m). DNA/protein residues and water molecules are depicted as in (*b*). A 1σ-contoured σ_A_-weighted 2*F*_o_ − *F*_c_ composite omit map of G*O_light_ is superposed over the DNA to highlight the presence of oG within the active site. (*e*) Detail of the exosite of G*O_light_ versus GO_K249Q_, shown as in (*d*). A calcium ion present in G*O_light_ but not in GO_K249Q_ is represented by a green sphere, with a 1σ-contoured σ_A_-weighted 2*F*_o_ − *F*_c_ composite omit map shown in orange.

Upon illumination (95 J) and cooling in liquid nitrogen, the oG*–hOgg1-N149C complex (G*O_light_, PDB entry 9kl8) underwent subtle (r.m.s.d. of 0.265 Å over 294 common C^α^ atoms between G*O_dark_ and G*O_light_) but significant conformational changes (Fig. 3 ▸ and Supplementary Fig. S5), which we analysed by generating *F*_o_(G*O_light_) − *F*_c_(G*O_dark_) difference electron-density (DED) maps [Figs. 3 ▸(*b*) and 3 ▸(*c*)]. While the Cys149 disulfide cross-link persisted in the complex [Supplementary Fig. S5(*c*)] and the upstream DNA branch remained dynamic in the cryotrapped structure, a strong DED feature appeared within the active site [Figs. 3 ▸(*b*), 0.264 e^−^ Å^−3^]. Comparison with the previously published, catalytically inert, oG–hOgg1-K249Q complex (GO_K249Q_; Shigdel *et al.*, 2020 ▸) revealed that the novel DED feature in G*O_light_ partially overlapped the active-site oG in GO_K249Q_ [Fig. 3 ▸(*d*)]. To us, this suggested that the uncaging reaction had been successful, and we refined oG partially occupying the hOgg1 active site (68% occupancy) and displacing water molecules B and C [Figs. 3 ▸(*b*) and 3 ▸(*d*)]. Meanwhile, the G*O_dark_ oG* pentose site had been completely vacated in G*O_light_, suggesting that photodeprotection had occurred quantitatively [Fig. 3 ▸(*a*) and Supplementary Fig. S5]. Interestingly, the resulting G*O_light_ complex has a markedly lower normalized *B* factor than G*O_dark_, a change that is centred around the DNA moiety (Table 1 ▸) and which results in better defined positions for oG and neighbouring bases [Fig. 3 ▸(*a*)]. In good agreement with a disorder-to-order transition, the DED maps are dominated by positive peaks, while negative peak are rare [Figs. 3 ▸(*b*) and 3 ▸(*c*)]. We propose that upon entering the active site, oG significantly increases the affinity of hOgg1 for the DNA, possibly including tighter binding by the G/P-rich loop, which also becomes better ordered in G*O_light_ [Fig. 3 ▸(*a*)]. Ordering of the complex due to binding of the DNA lesion correlates well with recent TRX studies on the post-repair dynamics of DNA repair by photolyase (Maestre-Reyna *et al.*, 2023 ▸), where lowered affinity for repaired thymines induced DNA disorder characterized by a DED dominated by negative peaks.

Compared with the GO_K249Q_ complex, the G*O_light_ active-site oG does not penetrate as deeply into the protein, with a 1.1 Å distance between the centres of mass of the oG moieties of the two superposed structures (r.m.s.d. of 0.449 Å over 262 common C^α^ atoms). Although it is conceivable that the DNA–protein cross-link may hinder full oG insertion into the active site by restricting its conformational freedom, several lines of evidence point towards this effect being rather unlikely. Firstly, previous studies have shown that tethered complexes are fully active (Lee *et al.*, 2008 ▸). Secondly, in both of our structures the distance between the cross-link partners, N4 of C^‡^ and S^γ^ of Cys149, remains constant (3.9 versus 4.2 Å), and much shorter than the extended, 5.4 Å long, tether. These two points suggest that despite potentially restricting conformational freedom, active conformations can still be adopted due to high tether flexibility.

In G*O_light_, oG makes contact via its NH_2_ group with the Asp268 side chain, while its O6 position interacts with a crystallographic water molecule lodged deep within the active site (water A in Fig. 3 ▸). By contrast, several key interactions of the GO_K249Q_ complex are missing in G*O_light_, *i.e.* π–π stacking between oG and Phe319 and a hydrogen bond between Gln315 and the oG N1 position. In fact, the overall side-chain geometry of the G*O_light_ active site is more reminiscent of that of G*O_dark_ than of the complex described by GO_K249Q_ [Fig. 3 ▸(*b*) versus 3 ▸(*d*)]. Taken together with the more distal position of oG, the active-site resemblance between G*O_dark_ and G*O_light_ suggests that the latter represents a late intermediate during base extrusion but prior to fully achieving the catalytically active conformation.

In good agreement with the interpretation of G*O_light_ as a base-extrusion intermediate, a strong DED peak (0.234 e^−^ Å^−3^) could be observed within the exosite of the structure [Fig. 3 ▸(*c*)]. Here, its spherical shape, the distinct conformations adopted by His270 and Asp268 in G*O_light_ [Figs. 3 ▸(*c*) and 3 ▸(*e*)] and the high concentration of CaCl_2_ in our crystallization condition (200 m*M*) led us to interpret it as a calcium ion coordinated by His270, Asp268 and the O4′ position of the oG deoxyribose moiety [Figs. 3 ▸(*c*) and 3 ▸(*e*)]. To us, this suggests that upon base extrusion, oG is accompanied by a calcium ion, which was prevented from further penetration into the active site by the exosite. In summary, comparison between G*O_light_, G*O_dark_ and previously published structures has revealed the feasibility of LT-TRX experiments based on the G*O_dark_ crystal form, as well as suggesting a secondary role for the exosite as an ion filter during base extrusion.

## 4. Conclusion

In the current work, we have assessed the potential of G*O_dark_ as a target for LT-TRX analysis. Although our conclusions are positive, *i.e.* the crystal production yield is sufficient and *in crystallo* reactivity could be determined with a high degree of confidence, it is worth considering the structural changes that could be observed in a potential G*O_dark_ LT-TRX study. Based on the G*O_light_ structure and previous analysis of the exosite (Banerjee *et al.*, 2005 ▸; Lee *et al.*, 2008 ▸), TRX analysis may reveal GIC structural intermediates and clarify the role of the exosite as a possible divalent-cation filter. Meanwhile, the mixed structural features of the G*O_light_ active site, *i.e.* partially oG-populated but with a G*O_dark_-like geometry, suggest that changes in oG–active site interactions are structurally resolvable. As cross-linking between DNA and protein was needed to obtain the low-affinity G*O complex, observing full complex release is out of the question. However, the observed illumination-dependent loss of diffraction power by G*O_dark_ suggests the enticing possibility of directly observing hOgg1-catalyzed DNA cleavage at the oG site, which leads to a single-strand break.

## 5. Related literature

The following references are cited in the supporting information for this article: Afonine *et al.* (2012 ▸), DeLano (2008 ▸), Emsley *et al.* (2010 ▸), Fromme *et al.* (2003 ▸), Kabsch (2010 ▸), Krissinel & Henrick (2007 ▸), Liebschner *et al.* (2019 ▸), McCoy *et al.* (2007 ▸), Rould & Carter (2003 ▸), Stein (2008 ▸), Winn *et al.* (2011 ▸) and Yamashita *et al.* (2018 ▸).

## Supplementary Material

PDB reference: human 8-oxoguanine glycosylase N149C mutant with DNA containing photocaged 8-oxoguanine, 9kky

PDB reference: after deprotection, 9kl8

Supporting information. DOI: 10.1107/S2052252525006062/mah5002sup1.pdf

## Figures and Tables

**Table 1:** Data-collection and refinement statistics Values in parentheses are for the highest resolution shell.

	G*O_dark_	G*O_light_
PDB code	9kky	9kl8
Data collection		
Space group	*P*6_5_22	*P*6_5_22
Wavelength (Å)	0.976254	0.976254
*a*, *b*, *c* (Å)	89.73, 89.73, 212.16	91.46, 91.46, 212.77
α, β, γ (°)	90, 90, 120	90, 90, 120
Resolution (Å)	30.00–2.65 (2.81–2.65)	42.01–2.48 (2.57–2.48)
Observed reflections	67308 (11625)	3063098 (474697)
Unique reflections	14869 (2389)	19518 (3051)
Multiplicity	4.5267 (4.866)	157 (156)
Completeness (%)	96.4 (99.5)	100.00 (100.00)
Mean *I*/σ(*I*)	9.92 (1.83)	16.76 (1.62)
CC_1/2_	0.992 (0.522)	0.998 (0.879)
Refinement		
Resolution (Å)	29.07–2.81 (2.91–2.81)	42.01–2.48 (2.57–2.48)
Unique reflections	12727 (1241)	19518 (3051)
Completeness (%)	98.06 (99.52)	100.00 (100.00)
*R*_work_	0.2232 (0.3198)	0.2127 (0.3423)
*R*_free_	0.2541 (0.3685)	0.2509 (0.3814)
R.m.s.d., bond lengths (Å)	0.008	0.002
R.m.s.d., angles (°)	1.2	0.48
Ramachandran favoured (%)	96.46	96.14
Ramachandran allowed (%)	3.54	3.86
Ramachandran outliers (%)	0	0
Rotamer outliers (%)	1.26	3.25
Clashscore	1.71	3.52
Average *B* factor (Å^2^)	81.86	70.99
Average protein *B* factor (Å^2^)	73.03	59.50
Average DNA *B* factor (Å^2^)	166.82	142.61

## Data Availability

All coordinate and reflection files are available from the Protein Data Bank under PDB codes 9kky (G*O_dark_) and 9kl8 (G*O_light_).
