# Supplementary material for: Revisiting the co-crystal structure of a DNA glycosylase with photocaged substrate: a suitable time-resolved crystallography target?
Source: IUCrJ. 2025 Jul 14;12(Pt 5):515–22. doi: 10.1107/S2052252525006062 (PMC12403168; doi:10.1107/S2052252525006062)
Supplement: Supplementary file 1 [file m-12-00515-sup1.pdf]

IUCrJ

Volume 12 (2025)

Supporting information for article:

Revisiting the co-crystal structure of a DNA glycosylase with photocaged substrate; a suitable time-resolved crystallography target?

Tomoki Imura, Yuhei Hosokawa, Kai-Chun Yang, Yuki Ban, Hsuan-Yu Shih, Junpei Yamamoto and Manuel Maestre-Reyna

S1. Materials and methods

S1.1. General

Reagents and solvents for solid-phase DNA synthesis were purchased from Glen research. All other reagents and solvents were purchased from either FUJIFILM Wako Pure Chemical Corporation, Tokyo Chemical Industry, Nacalai Tesque, or Hampton Research. HPLC analyses were carried out on a Gilson gradient-type analytical system equipped with a Waters 2998 photodiode-array detector. A Waters μBondasphere C18 5 μm 300 Å column (3.9 x 150 mm) was used, at a flow rate of 1.0 mL/min, with a linear gradient of acetonitrile in 0.1 M triethylammonium acetate (pH 7.0) generated over 20 min. Matrix-assisted laser desorption/ionization time-of-flight mass spectrometry (MALDI TOF MS) was performed with a Bruker ultraflex III in the negative ion mode using 3-hydroxypicolinic acid as a matrix.

S1.2. Preparation of oligonucleotides

Protected phosphoramidite building blocks of the caged 8-oxo-2'-deoxyguanosine (oG*) and the 2'-deoxy-4-triazolouridine (U^{tri}) were synthesized according to the reports (Lee *et. al.,* 2008). Oligonucleotides containing oG* and U^{tri} were synthesized on an Applied Biosystems 3400 DNA synthesizer, using the phosphoramidite building blocks of oG*, U^{tri}, and protected deoxyribonucleosides for ultra-mild DNA synthesis (Glen Research), which were assembled on the solid support. The damaged strand, d(AGCGTCCAoG*GTCTACC), was cleaved from the support by treatment with 28% ammonia water at room temperature for 1 h and deprotected by heating at 55°C for 4 h, while the complementary strand, d(TGGTAGACU^{tri}TGGACGC), was cleaved from the support by treatment with 3 M cystamine solution at room temperature overnight. Upon the treatment, U^{tri} was converted to an N^4-modified cytosine ($C^‡$), yielding the disulfide crosslinking oligonucleotide, d(TGGTAGAC$C^‡$TGGACGC). The products were purified by HPLC with an acetonitrile gradient of either 6–14% or 7–15%, and analyzed by MALDI TOF MS (Entries 3 and 5 in Table S1). Oligonucleotides without these modifications, d(AGCGTCCAGGTCTACC) and d(TGGTAGACCTGGACGC), were also synthesized and purified in the same way as described.

Photodeprotection of the oligonucleotide containing oG* was examined using a CL-1501 LED emission system (Asahi Spectra Co., Ltd.) equipped with a CL-H1-365-9-1-B LED head unit and a CL-P2-54 light-guiding quarts tip. Aliquots (10 μL) of the single-stranded oligonucleotide containing oG* (20 μM) in 1.5 mL tubes were illuminated with 365 nm light at about 5 mm above the surface of the solution for 0.5–5 min, and the mixtures after illumination were analyzed by HPLC using the 7–15% acetonitrile gradient. The peak emerging at a retention time of 10 min in the chromatograms (Fig. S1) was purified with HPLC and analyzed by MALDI TOF MS (Entry 2 in Table S1),

confirming the successful deprotection of oG* to yield oG. The purified product, d(AGCGTCCAoGGTCTACC), was used for biochemical assays. This entire synthesis and purification procedure had an overall yield of between 30 to 40%.

S1.3. Protein production

The codon-optimized cDNA of wildtype human 8-oxoguanine DNA glycosylase (hOgg1) was purchased from Azenta, and a part of the cDNA coding from Met12 to Arg327 was subcloned into a pET28a vector linearized with the NdeI and XhoI restriction enzymes. N149C mutation was introduced by PCR and In-fusion HD cloning kit (TAKARA). After sequencing the obtained plasmids, hOgg1 proteins were obtained in *Escherichia coli* with autoinduction. *E. coli* BL21(DE3) star competent cells were transformed with the plasmids and cultured at 37°C in 500 mL of Luria-Broth (LB) medium supplemented with 20 µg/mL kanamycin. When OD at 600 nm reached 0.2, the culture was cooled to 25°C by changing the temperature of the shaker, and further shaken for 24 h. The cells were harvested by centrifugation, frozen in liquid nitrogen, and stored at –80°C. The pellet was thawed on ice, resuspended in lysis buffer (20 mM Tris-HCl, 50 mM NaCl, and 5% glycerol, pH 7.5), and lysed via three cycles of mechanical cell disruption (constant systems cell disruptor). After centrifugation of the lysate, the His-tagged proteins were successively purified with TALON Metal Affinity Resin (Clontech) and a HiTrap Heparin HP column (Cytiva). The obtained protein solution was buffer-exchanged to lysis buffer and stored at –80°C, with a total yield of 10 to 20 mg of purified protein per L of culture.

S1.4. Biochemical assays

Oligonucleotides d(AGCGTCCAXGTCTACC), where X represents either G, oG, or oG*, were treated with T4 polynucleotide kinase (TAKARA) in the presence of γ-^{32}P-ATP (~200 kBq, Levvity Healthcare Sciences, Inc.), at 37°C for 30 min. The 5'-^{32}P-labeled oligonucleotides were passed through a G-25 Microspin column (Cytiva), and hybridized to the complementary strand, d(TGGTAGACCTGGACGC). To check the enzymatic activity of the recombinant hOgg1 proteins, the duplexes (400 fmol) were incubated with 0–2 molar equivalents of hOgg1 proteins at 37°C for 1 h, in buffer containing 20 mM Tris-HCl, 50 mM NaCl, 2 mM DTT, 0.24 mg/mL bovine serum albumin, and 5% glycerol, pH 7.5. The reaction was quenched by addition of 5 N NaOH (2 µL) and heat at 85°C for 5 min. After further addition of denaturing solution (10 µL; 95% formamide, 20 mM EDTA, 0.025% bromophenol blue and 0.025% xylene cyanol), the reaction products were separated by electrophoresis on a 20% polyacrylamide/7.5 M urea gel. The dried gels were analyzed with an FLA7000 image analyzer (GE Healthcare). For the caged duplex, reaction of hOgg1 proteins after the uncaging was also examined. The reaction mixtures were illuminated with the 365 nm LED for 30 s and then incubated at 37°C for 1 h. The products were analyzed in the same way as described.

S1.5. DNA detection in co-crystals

To justify the presence of DNA in the obtained co-crystals, several crystals kept in dark for a month were dissolved in water in the presence of 2-mercaptoethanol to cleave the disulfide crosslinking between protein and DNA. After removal of proteins by phenol/chloroform treatment, oligonucleotides were recovered by ethanol precipitation. The residues were dissolved in water, and analyzed by HPLC using 5–17% acetonitrile gradient generated over 30 min.

S1.6. Data collection

Data collection took place at the National Synchrotron Radiation Research Center, Taiwan Photon Source beamline 07A. Crystals were transferred in the dark to the synchrotron facility, where G*O_{dark} crystals were mounted onto nylon loops under safety light (635 nm), soaked with crystallization solution supplemented with 30% glycerol for 10 s (cryobuffer), exposed to air for 10 seconds, flash frozen in liquid nitrogen, and finally mounted on the beamline's goniometer head. To determine crystal photosensitivity, crystals were first fragmented with a microtube homogenizer (Fisherbrand Pellet pestle cordless motor, Fisher Scientific) for a total of 80 seconds separated by a 40 s on-ice incubation period. Fragmented crystals were then soaked in cryobuffer for 10 seconds, loaded on a SwissCI SWISSMX CM Support holder and then exposed for 10 seconds to a microscope halogen lamp equipped with an U-FUW filter (Olympus, 340-390 nm pass through range). To prevent thermal effects, light was transmitted to the crystals via a 7 mm diameter optic fibre. The tip of the optic fibre was fixed 50 mm away from the loop, which resulted in an illuminated area of ~64 cm^2 at the loop position. Energy dose was regulated by adjusting nominal power output of the microscope light via a built-in attenuator. The light source had a maximum nominal power output of 130 W, which could be attenuated in steps of ~1%. Thus, the nominal energy output of the light-source as shown in Fig. 2d was calculated by multiplying nominal output of 130 W by the 10 second exposure time and the percentage of transmission (100% - percentage of attenuation). As a control, lysozyme crystals were also grown and optimized to have a similar size to G*O crystals (crystallization conditions: 15 mg/mL lysozyme, 24% w/v NaCl, 8% w/v PEG6000, 0.1 M sodium acetate pH 4.5). These were then treated as G*O crystals (both light and dark). To obtain a complete G*O_{light} dataset, G*O_{dark} fragmented crystals were loaded on a SwissCI SWISSMX CM Support holder, illuminated as above with a 6% percentage of transmission, and flash frozen in liquid nitrogen. Data collection followed immediately using custom software at the TPS07A beamline.

S1.7. Data processing

The G*O_{dark} dataset was processed with XDS (Kabsch, 2010) and scaled with scala, a piece of software from the ccp4i suite (Winn *et al.*, 2011). Similarly to previous hOgg1-DNA co-crystal structures portraying intermediates or other high energy states (Banerjee *et al.*, 2005; Fromme *et al.*, 2003), G*O_{light} datasets derived from individual crystals presented low completeness. To address this

issue, we prepared crystal fragments which could then be collected via a fixed-target serial approach followed by processing with KAMO/XDS (Yamashita *et al.*, 2018; Kabsch, 2010). The resulting G*O_{light} composite dataset had very high completeness and multiplicity, reaching a resolution of 2.48 Å. Lysozyme and other illuminated crystals were processed by XDS, but only to obtain maximum resolution statistics, as shown in Fig. 2d.

S1.8. Solution and refinement

The processed G*O_{dark} dataset was solved by molecular replacement with phaser (McCoy *et al.*, 2007) with a c-beta stripped-down (Stein, 2008) version of GIC (PDB code 1YQR) (Banerjee *et al.*, 2005) as the search model. G*O_{light} was also solved by molecular replacement; however, the search model was the refined G*O_{dark} structure.

Structural models were produced via a combination of automated and manual refinement with phenix.refine and coot (Afonine *et al.*, 2012; Emsley *et al.*, 2010).

S1.9. Structure analysis and visualization

Despite low isomorphism between dark and light dataset (overall correlation coefficient of 0.70 and R_{iso} of 31.8%) Fo-Fo isomorphous density maps (Rould & Carter, 2003) were generated by the phenix tool of the same name using the G*O_{dark} structure for phasing. Fo-Fc maps were generated by three cycles of rigid body refinement of the G*O_{light} dataset against the G*O_{dark} coordinates via phenix.refine (Liebschner *et al.*, 2019). Composite omit maps were generated by the phenix tool of the same name. Structural figures were generated with pymol (Delano, 2008). Analysis of crystal contacts was performed with PDBePISA (Krissinel E. & Henrick & Krissinel K., 2007).

Table S1 MALDI-TOF MS results of oligonucleotides used in this study

Entry	Sequence (5'→3')	Observed *m/z*	Calculated *m/z*[a]
1	d(AGCGTCCA**G**GTCTACC)	4841.180	4839.840
2	d(AGCGTCCA**oG**GTCTACC)	4857.174	4855.834
3	d(AGCGTCCA**oG***GTCTACC)	5020.355	5018.898
4	d(TGGTAGAC**C**TGGACGC)	4923.074	4921.230
5	d(TGGTAGAC**C**‡TGGACGC)	5062.946	5056.473

[a]Calculated in $[M–H]^-$ forms

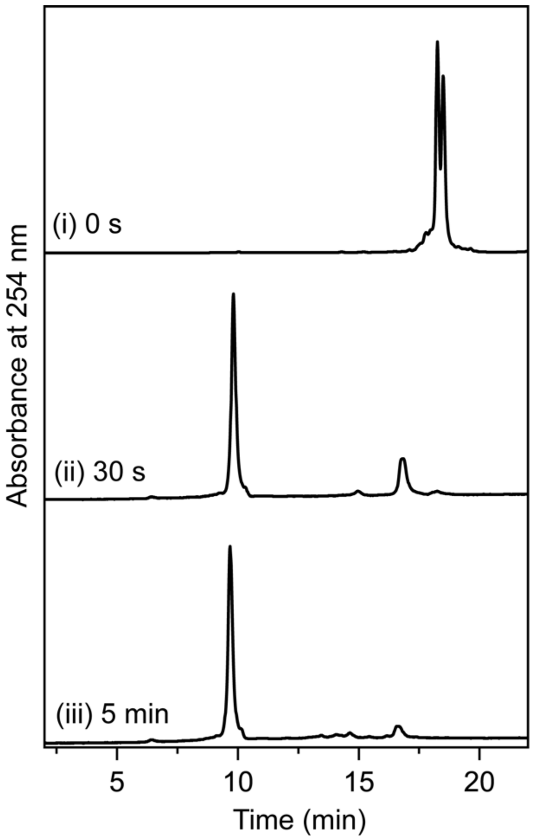

Figure S1 Uncaging of 8-oxoguanine. The oligonucleotide containing oG* was illuminated with the 365 nm for (i) 0 s, (ii) 30 s, and (iii) 5 min, and the reaction was monitored by HPLC. The product emerging at a retention time of about 10 min was purified and analyzed by MALDI TOF MS, and the obtained *m/z* value (Entry 2 in Table S1) was identical to calculated *m/z* value for the oG-containing oligonucleotide.

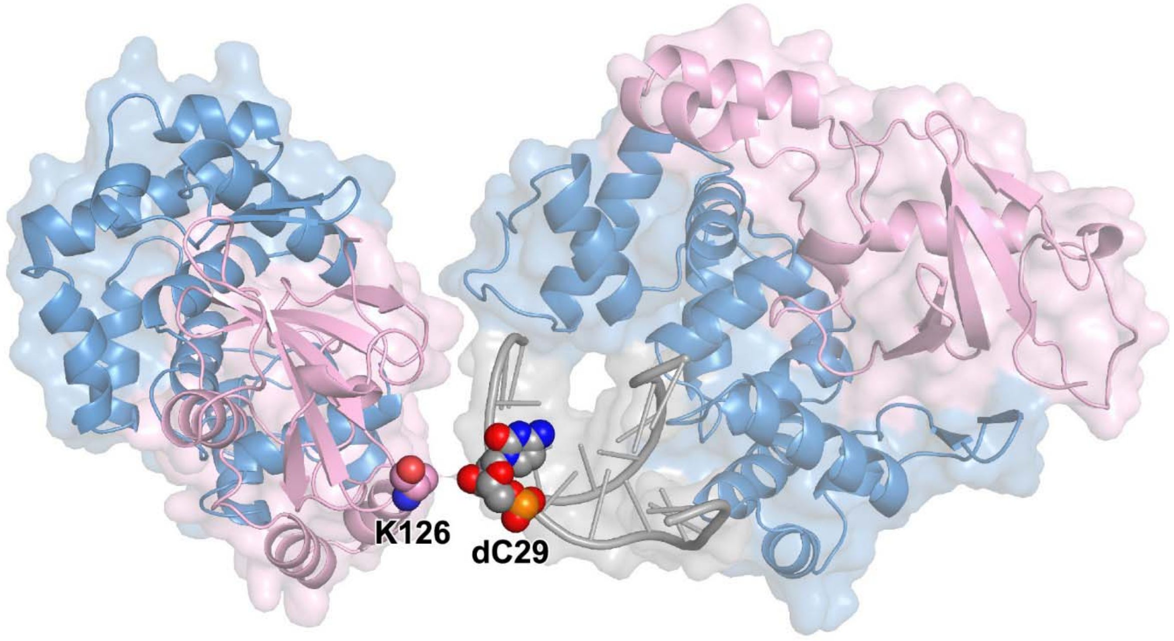

Figure S2 DNA-mediated crystal contacts in G*O$_{dark}$. Cartoon representations depict two neighbouring crystallographic asymmetric units (ASU). Each ASU is composed of one hOgg1 molecule (pink and blue) bound to the oG* containing DNA duplex (grey). Supramolecular assembly analysis via PDBePISA revealed a crystal contact mediated between dC29 of one ASU and K126 of the neighbouring one. Both elements involved in the crystal contact are shown as spheres.

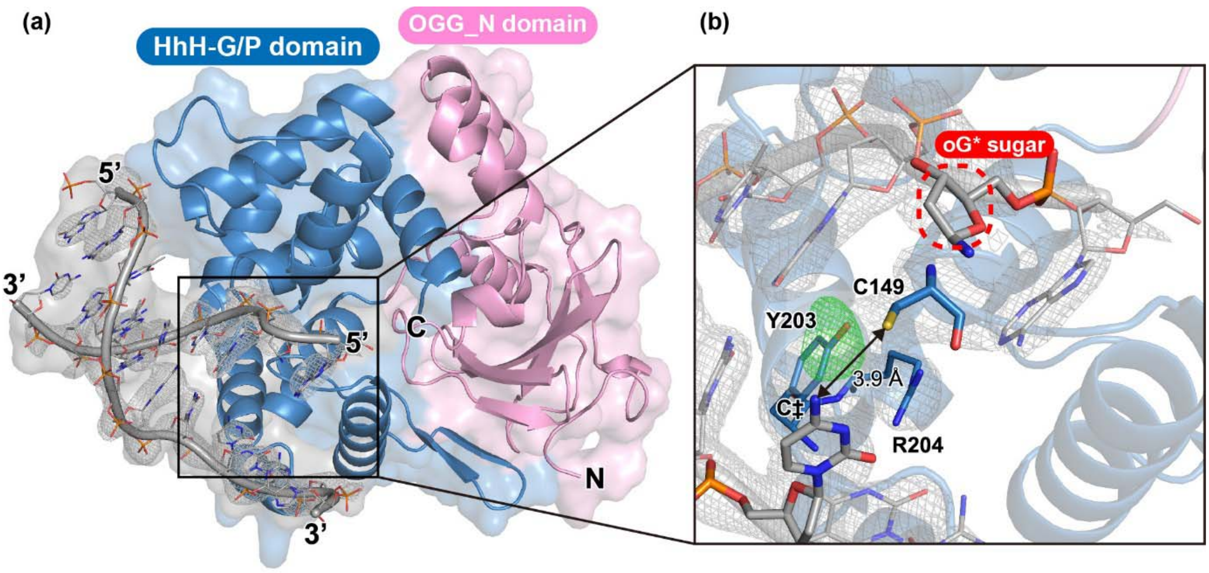

Figure S3 General overview of the G*O$_{dark}$ complex. (a) Cartoon representation of the G*O$_{dark}$ complex, with the hOgg1 HhH-G/P and OGG_N domains in blue and pink, respectively. The DNA duplex (grey) is shown both as a cartoon and stick representation. A 1σ-contoured sigmaA weighted 2Fo-Fc map overlays the DNA region (grey). To showcase the similarities between G*O$_{dark}$ and the GIC (1YQK), a surface representation of the latter is superposed over G*O$_{dark}$. (b) Detail of the lesion binding site in G*O$_{dark}$. Structural features are depicted as in (a), while a red circle stresses the presence of the oG* sugar, but the absence of its base moiety. Putative linker position is highlighted by a black double pointed arrow and by a sigma weighted 3σ contoured Fo-Fc DED map.

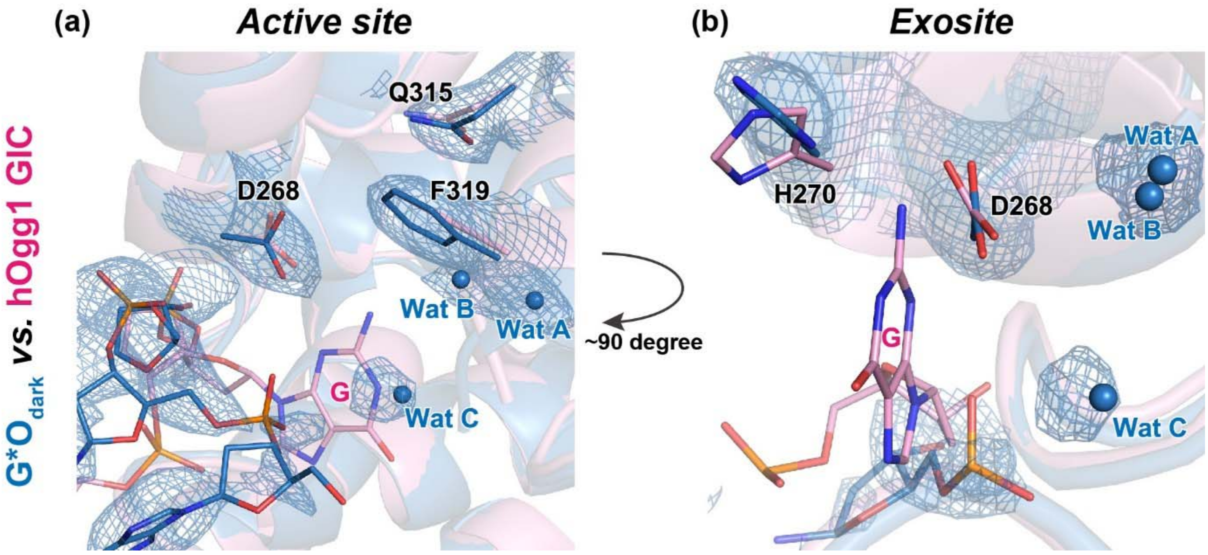

Figure S4 Details of active site and exosite of G*O$_{dark}$ vs. GIC. (a) Details of active site of G*O$_{dark}$ (blue) versus the previously published G-interrogating complex (pink, 1YQK). DNA/protein residues are shown as stick diagrams, while water molecules corresponding to G*O$_{dark}$ as spheres. A 1σ-contoured sigmaA weighted 2Fo-Fc map of G*O$_{dark}$ is superposed over the structural models. (b) Detail of exosite of G*O$_{dark}$ versus the previously published G-interrogating complex, shown as in (a).

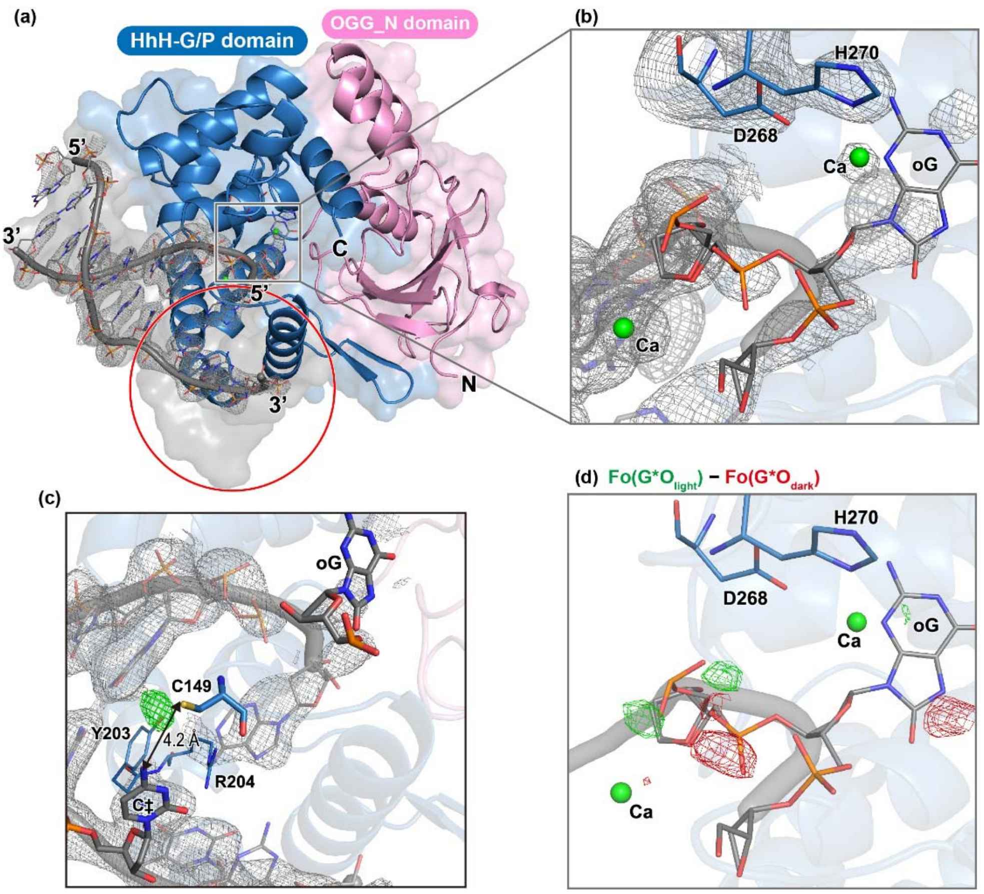

Figure S5 General overview of the G^*O_{light} complex. (a) Cartoon representation of the G^*O_{light} complex, with the hOgg1 HhH-G/P and OGG_N domains in blue and pink, respectively. The DNA duplex (grey) is shown both as a cartoon and stick representation. A 1σ-contoured sigmaA weighted 2Fo-Fc map overlays the DNA region (grey). To showcase the similarities and disparities between G^*O_{light} and GO_{K249Q} (6W0M), a surface representation of the latter is superposed over G^*O_{light}. Note the significant differences in DNA geometry between the two structures, which results in significant portions of the grey GO_{K249Q} surface representation appearing empty in the figure (red circle) (b) Detail of the lesion binding site in G^*O_{light}. Structural features are depicted as in (a). Associated calcium ions are depicted as green spheres. (c) Detail of the DNA-protein linker region shown as in (a). Putative linker position is highlighted by a black double pointed arrow and by a sigma weighted 3σ contoured Fo-Fc DED map. (d) 2.5 σ contoured isomorphous difference density map (G^*O_{light}-G^*O_{dark}) generated by the phenix tool of the same name. Due to low isomorphism of the datasets the map is of rather poor quality. However, it is possible to see that some positions occupied by the G^*O_{dark} DNA backbone overlap with DED peaks (red for negative, green for positive)
